# Modeling Mentor-Mentee Dialogues in Film

**DOI:** 10.1080/01969722.2018.1556438

**Published:** 2019-02-20

**Authors:** Anna Dobrosovestnova, Marcin Skowron, Sabine Payr, Robert Trappl

**Affiliations:** Austrian Research Institute for Artificial Intelligence, Vienna, Austria

**Keywords:** Conversation analysis, dialogue management and generation, mentoring

## Abstract

With a view to inform the design of a mentor-like synthetic agent that is to engage in a coherent and consistent in character conversation with human subjects, we conducted a data-driven analysis of verbal communication between fictional mentor and mentee characters in films. While in our earlier work the focus was on the conversation strategies of mentor characters, here we present the extended model, wherein conversation activity of both mentor and mentee characters is accounted for. To examine and to formalize local communication actions and extended goals that the two characters achieve jointly, categories of intents, projects and relationship phases were introduced. The resulting annotated corpus of mentor and mentee characters’ utterances was analyzed qualitatively and quantitatively. In furtherance of the automated in-character dialogue generation task, a range of the state-of-the-art approaches to automated utterances classification was evaluated.

## Introduction

Always two there are, no more, no less. A master and an apprentice.

Master Yoda

This paper introduces the extended model of strategic talk in film developed to inform the design of an automated dialogue generation system that is to engage in conversations with human users in character. Character of mentor was chosen as a prototype as it exhibits a wide range of functional and psycho-social characteristics which are relevant in light of the rapidly developing sectors of online tutoring (Kulik and Fletcher 2016) and educational robotics (Edwards and Cheok 2018).

Our earlier model of strategic talk in film (Payr et al. 2017) was put forward based on the annotations and analysis of the corpus of mentor characters’ utterances from a sample of 45 films. We chose to turn to film scripts because it is hard to come by natural mentor–mentee conversations corpora. It is our assumption that, despite their artificiality and objectives that diverge from those normally found in natural conversations e.g. advancing the plot, viewer engagement etc., scripted dialogues still follow the fundamental logic of conversations (Grice 1975).

The model presented here extends our earlier analysis to include the utterances of mentee characters and introduces three new structural categories. The reason for the incorporation of mentee characters’ utterances is twofold. Firstly, conversations are temporal and bi-directional: what a conversation participant says is informed by what was said in the conversation previously and what kind of effect they expect an utterance will have on the conversation partner (Levinson 2013). Our objective behind the extended model was to preserve and reflect on the collaborative nature of communication. Secondly, in order for the dialogue generation system to be able to generate meaningful and consistent responses to user inputs, it is desirable that the system can classify user inputs not only in terms of their semantic theme, but also in terms of the communicative action they perform in a given context, for example, whether an utterance is intended as an explanation or an instruction.

The questions that shaped our research were as follows: i) What kind of typical communication actions (Schegloff 2007) mentor and mentee characters exhibit at the level of a single utterance/turn? ii) What are the defining processes, events and phases that a mentoring relationship undergoes? iii) How are these processes expressed in language? iv) Whether some meaningful structural correspondences between local and global structural units can be established? and finally, v) How does the automated classification of utterances compare to that of a human expert?

To explore these themes, three categories operating at micro-, meso- and macro-levels of scripted conversations between mentors and mentees were introduced. The category of *intents*, inspired by conversation analysis approach (Elliott, Hoey and Kendrick 2017), was introduced to classify mentor and mentee characters’ typical conversation actions (e.g. explain, ask, scold, etc.) at the level of a single utterance/turn. The category of *projects* was developed to represent extended sequences of mentor and mentee intents that shape into more global processes typical of mentoring relationships e.g. training, bonding, etc. Finally, the category of *mentoring relationship phases* was adopted from literature on mentoring (Kram 1983) to provide a general framework for the exploration of how sequences of intents and projects map onto the global dynamics of mentoring relationship.

Bearing in mind that the objective we are pursuing is to design a dialogue system that relies on recognition and generation of written text, the model we propose does not incorporate other communication modalities (e.g. body language, prosody, pauses) that otherwise play an important role in natural conversations.

The structure of the paper is as following. In Methods, we outline the strategy that we used to compile, annotate, and analyze a corpus of utterances from scripted dialogues between mentor and mentee characters in film. In Results, we provide an overview of the quantitative analysis of the annotated corpus and related discussion. Finally, in Classification we evaluate the performance of the automatic classifier against that of a human expert. The paper concludes with a review of the limitations of our approach and explores possible directions for future research.

## Methods

### Data Set

Mentor–mentee dialogues from Matrix (1999) and Kung Fu Panda (2008) were extracted from the Internet Movie Script Database (IMsDB)^1^ and automatically broken down to scenes and unique utterances. Similar procedure was repeated for Chasing Mavericks (2012). The film was extracted from Springfield! Springfield! website for film scripts^2^ and broken to scenes manually due to the source format limitations. The three films mentioned were chosen as they portray the evolution of the lead mentor-mentee relationships, from the first encounter to the termination/redefinition.

Traditionally, mentoring is defined as a dyadic relationship in which mentor – a person in age or experience – provides guidance and support to a less experienced protege (Ensher et al. 2001). In line with conversation analysis approach, mentoring can also be thought of as an activity type, wherein its principal participants are goal-oriented, socially-constituted, and bounded (Drew and Heritage 1992). Despite an apparent straightforwardness of this definition, it is not always easy to delineate communication activities that fall under one or another activity type (Levinson 1992). This is also true of film scripts, where interactions between mentor and mentee characters incorporate a wide range of activities and phases, some of which extend beyond the framework of mentorship. Earlier research suggests that the main functions provided by mentors span across two principal dimensions: i) career/skills-oriented or instrumental (coaching mentees, sponsoring career advancement, providing challenging assignments) and ii) psycho-social (building trust, interpersonal bond, enhancing mentee’s personal growth and self-worth) (Ragins and Kram 2007). The final data set was compiled in agreement with this definition. Namely, the scenes, where both mentor and mentee characters are present but the nature of their interactions does not explicitly fall under neither instrumental nor psycho-social dimensions were excluded. The scenes, where mentor and mentee characters interact with support characters were also excluded, unless specified otherwise (i.e. support characters Oogway and Tai Lung were preserved for the reasons we will address further). The final data set (Training set) thus compiled totals 1193 utterances.

### Intents

Drew and Heritage (1992) suggest that an activity type will consist of a range of ordered sequences of local communication actions or, as we refer to them, *intents*. The kinds of actions performed and the order of these will be constrained by the activity type in question. For example, for mentoring activity type, it is reasonable to assume that activities such as asking, explaining, evaluating will be featured. Hence, the task we pursued when developing labels for the Intents category was to identify and name communication actions typically manifested in mentor-mentee dialogues. Following Schegloff (2007), we started from individual instances of data in their embedded context and asked ourselves what a character appeared to be doing when uttering a phrase, also in respect to the effect that the phrase has on the response that follows. Note that in scripted dialogues, an utterance will also perform functions that are meant to have an effect on spectator. Our interest, however, was solely on the communication acts as directed at and acted upon other characters. Thus, extradiegetic functions inscribed into characters’ utterances by scriptwriters were ignored. As result of iteratively going through the data set, 22 labels for mentor intents (Table 1) and 21 labels for mentee intents were established (Table 2).

**Table 1.** Mentor intents.

Intent	Explanation	Examples
1 Seek out	Mentor’s direct or indirect invitation to join the training/quest	“I can turn you into the Dragon Warrior!”, “Do you want to know what it is, Neo?”
2 Profile	Mentor’s questions aimed at exploring mentee’s background, values, prior experiences and interest towards the quest	“Can you tell me, Neo, why are you here?”, “How old are you? What, 6, 7?”
3 Social	Salutations, social pleasantries, small talk	“How’s it going over there?” “Get some rest.”
4 Set the rules	Mentor setting the terms and the rules that the mentee is to follow if he/she agrees to take part in the mentoring process	“After this, there is no going back.”, “When you have been trained, you may eat.” l teach you what you need to know, and it’s over.”
5 Single out	Mentor acknowledging mentee’s talent/gift	“The universe has brought us the Dragon Warrior!”, “You are here because you have the gift.”, “Now, l know how good you are. l’ve seen you out there.”
6 Confirm	Mentor responding positively to preceding utterance/question from mentee	“Yes”, “Alright”, “Ok”
7 Problem setting	Mentor announcing a quest-related problem/challenge that needs to be addressed	“I have had a vision Tai Lung will return.”, “We have to do something”
8 Encourage	Mentor encouraging mentee to continue the training	“You can make it.”, “The answer is coming”, “Oh, there’s a way, all right.”, “A real warrior never quits!”
9 Support	Offer of emotional support and friendship; acknowledging emotional needs of mentee	“I imagine, right now, you must be feeling a bit like Alice, tumbling down the rabbit hole?”, “I understand [how you feel].”
10 Explain	Narrating the facts about the story-world; sharing words of wisdom to guide mentee in construing their model of the world and acquiring the knowledge necessary to operate within it	“The Matrix is everywhere, it’s all around us, here even in this room.”, “Your mind is like this water, my friend. When it is agitated, it becomes difficult to see.”, “Now, big-wave surfing, that’s a different ball game. That’s about how you perform when everything goes wrong.”
11 Instruct	Mentor providing explicit instructions to action; Can be paraphrased as ”do/don’t do this”.	“So you’re gonna be doing this from now on, every day, 1 2 weeks.”, “You must empty yourself to free your mind.”, “Focus.”
12 Ask	Questions about the training process and the quest	“Do you believe me now?”, “You want to know why?”
13 Seek confirmation	Seeking mentee’s confirmation that the information presented was heard and understood	“ls that clear?”, “Did you hear what l just said?”
14 Apologize	Mentor acknowledging their mistake/wrongdoing and asking for mentee’s forgiveness	“I feel that I owe you an apology.”, “But perhaps that is my fault.”
15 Confusion	Expressions of confusion/lack of understanding of the situation	“I don’t understand.”
16 Evaluate	Providing positive or negative feedback	“Good”, “But your weakness isn’t your technique.”, “Wrong!”, “Look at you this fat butt.”
17 Test	Mentor challenging or provoking mentee	“Your next opponent will be me.”, “You don’t know? Or you don’t wanna know?”
18 Scold	Mentor telling mentee off or sharing their dissatisfaction	“Well, you just used up your entire allotment of dumb luck.”, “My patience is wearing thin.”, “You actually thought you could learn to do a full split in one night?”
19 Dismiss	Mentor denying mentee the right to be trained/participate in the quest	“You are not the Dragon Warrior.”, “And I am no longer your master.”, “Heh. Come on, get out of here. l got work to do.”
20 Share	Mentor sharing their personal story or emotional state	“Used to bob up and down like a cork just….Just to feel it. The abyss”, “…l love you no matter what.”
21 Depart	Mentor exiting mentoring relationship when they think mentee is ready to take over, or when they feel that there is nothing else they can do to help mentee to improve	“My time has come.”, “It is time for you to continue your journey without me.”
22 Volunteer	Mentor takes on a challenging task; in some cases means they might sacrifice themselves to save others	“I can hold him off long enough for everyone to escape.”, “This battle is between you [the evil character] and me.”

**Table 2.** Mentee intents.

Intent	Explanation	Examples
1 Seek out	Mentee’s attempt at initiating mentoring relationship; asking to be trained	“I need your help, Master.”, “Train me, then. Train me to ride it.”
2 Ask	Mentee’s seeking more information about the task/quest	“What truth?”, “How big was that wave?”
3 Doubt	Expressing doubts regarding their abilities to succeed in the quest	“I can’t do this!”, “I don’t believe it!”, “There’s no way to paddle out there especially with long boards.”
4 Resist	Showing resistance towards mentor and/or the quest; refusals to continue	“l’m not doing it again.”, “Why are you doing this to me?”, “I want out!”, “From the first moment I got here, you’ve been trying to get rid of me.”
5 Apologize	Apologizing for a mistake or mishap	“Sorry”, “I should’ve come to see you first.”, “Yes, sir. Sorry, sir”
6 Seek encouragement	Seeking mentor’s support	“You really believe I’m ready?”, “But what if l don’t have the strength, Jay?”
7 Volunteer	Volunteering for a challenge/task	“I’m gonna stop Tai”, “No, no, no! Give me my board!”
8 Self-affirm	Asserting their abilities/power	“l can handle it.”, “Get ready to feel the thunder.”, “Come and get it.”
9 Support	Providing emotional support to their mentor	“lf you look hard enough, there’s always a way through it.”
10 Excitement	Expressing excitement during the training or in the face of a challenge	“Man, did you see that? lt’s building! That was the longest one yet.”, “Would you look at this place!”, “Whoa!”, “That’s cool!”
11 Surprise	Expressions of surprise following newly discovered facts or learned skills	“I thought it wasn’t real.”, “Whaa?”
12 Report	Sharing observations about the world or aspects of the training/quest	“Looking like the best way out is to ride the current inside the rocks.”, “Feeling a little nauseous.”, “You You’re too fast.”
13 Confusion	Expressing confusion about the information received or the current state of the world	“l’m not sure l understand, sir.”, “I don’t I don’t understand.”
14 Share	Sharing their personal story and emotional experiences	“But it could never hurt more than it did every day of my life just being me.”, “l’ve been thinking about a lot of things lately.”, “Truth is that I am afraid.”
15 Praise	Expressing admiration for the mentor	“Yeah, Frosty!”, “I stayed because I thought if anyone could change me, could make me not me, it was you.”, “You’re Morpheus, you’re a legend.”, “It’s an honor.”
16 Social	Salutations, small talk, pleasantries	“Hey, how’s it going?”, “Huh Good morning, Master!”, ”Thanks.”
17 Confirm	Agreement/display of understanding of the information presented by the mentor	“Got it.”, “Yes, sir.”, “Deal”, “Sure.”
18 Respond	Responding mentor’s questions	“Because I don’t like the idea that I’m not in control of my life.”, “I told you I don’t believe in fate.”, “l mean, from books and maps. You told me to study the currents and the tides.”
19 Reinforce	Repeating mentor’s words or the newly learned information	“Deep breaths. Steady rhythm. Drive and glide.”, “Free my mind.”
20 Challenge	Confronting the mentor	“But when Oogway said otherwise, what did you do?”, “Tell me!”
21 Gratitude	Expressing gratitude	“You taught me to surf. l never thanked you for that.”, “Thank you.”

Once the labels for the intents category were finalized, the data set was annotated manually by a human expert. At this stage, a number of concessions and self-imposed restrictions had to be made. Both in natural and in scripted conversations, an utterance will often perform more than just one action (Levinson 1981; Schegloff 2007). For example, the utterance “*Stronger by the day, huh*?” delivered by Frosty, the mentor character from Chasing Mavericks, in response to the mentee expressing eagerness to surf a challenging break, takes the grammatical form of a question while at the same time functioning as an evaluation – “you are not strong enough” – and dismissal suggested by the sarcastic “huh”. Similarly, instructions will frequently overlap with explanations. For example, “*If you can free your mind, the body will follow*.” uttered by Morpheus (Matrix) is both a directive to free oneself of the preexisting assumptions (Instruct) and a lesson about the unity of body and mind (Explain). Furthermore, the interpretation of an utterance will depend on what precedes it in the conversation – the phenomenon referred to as cumulative effect (Levinson and Torreira 2015) – as well as other contextual parameters involved e.g. what we know about the character’s personality, how far in the relationship the two characters are etc. We will return to the data-grounded evaluation of the role of contextual parameters and metafeatures in the section on automated classification. That said, our approach when manually annotating for the intents category was to assign labels based on the dominant action that an utterance performs in a given context. Lastly, the boundaries of an intent as a unit of conversation do not necessarily coincide with the boundaries of an utterance and can span across several utterances. The format of the automated extracts dictated the necessity of the annotation for intent labels at the utterance level. However, sequences of identical intents are accessible as coherent chunks in the data file which allowed for further analysis.

### Adjacency Pairs

In view of our extended goal of the automated dialogue generation, we considered it useful to establish the types of adjacency pairs manifested in the dialogues between mentor and mentee characters. Schegloff (2007) defines *basic minimal adjacency pair* as an organizational conversation unit that, in unexpanded form, is 1) composed of two turns by different speakers, 2) adjacently placed, 3) relatively ordered (i.e. can be differentiated into “first pair part” and “second pair part”), and 4) pair-related, which means that not every second pair part can properly match the first pair part. In that regard, a first pair part will perform both an operation of retrospective understanding of the prior conversation and a prospective operation that makes relevant only a limited set of possible second pair parts.

It was agreed that adjacency pairs will be annotated manually by a human annotator following (Schegloff 2007). In conversations, the boundaries of a turn do not necessarily coincide with the boundaries of a single utterance/intent, that is one turn can be composed of several consequent utterances/intents by the same speaker (Levinson 2016). This is also true of some turns constituting parts of adjacency pairs in our data set. At the annotation stage, in cases when the intents constituting a character’s turn bear the same label (e.g. Explain-Explain), the given character’s turn was compressed to one label (Explain). Otherwise, if the turn constituting an adjacency pair part consisted of a sequence of different intents (e.g. Explain-Instruct), all labels were preserved, which resulted in an adjacency pair consisting of three or more intents in total.

For more detailed overview of the resulting adjacency pairs see Table 3.

### Projects

In natural conversations, activities often require contribution by all participants and span across boundaries of a single utterance/turn (Robinson 2013). This is certainly true of mentoring, wherein mentees are not passive recipients of mentors’ wisdom but are actively engaged in reciprocal reflection, questioning and problem-solving (Pfund et al. 2016). To explore the collaborative and dyadic nature of mentoring relationship, and how it is expressed in the dialogue structures, the category of projects was introduced. This category is meant to capture the pivotal processes and events e.g. Training, Bonding, Conflict, etc. that take place throughout mentoring relationships.

Here we relied on thematic analysis approach (Braun and Clarke 2006) to iteratively code the data set for different types of projects, which resulted in 14 project labels. Two important distinctions between intents and projects can be summarized as the following: i) if intents are annotated on the level of a single utterance/turn, projects by definition will incorporate communication acts by both mentor and mentee characters and ii) as mentioned previously, the category of intents has its roots in conversation analysis approach, where it is assumed that activities are recognized and understood (i.e. acted upon) by the participants of a conversation (Elliott, Hoey, and Kendrick 2017; Schegloff 2007). Projects, on the other hand, are an abstracted category that we introduce to make sense of the data in reference to the question posed. In other words, if the dialogues from our data set were enacted in real life, it would not follow that their participants would be necessarily aware that they are carrying out some sort of a project.

The following section provides an overview of the project labels we developed, accompanied by a brief description.

- Encounter: mentor and mentee characters meet for the first time. An encounter is characterized by the social interaction that will commonly incorporate profiling i.e. characters exchanging questions and answers with a purpose of acquiring more information about one another.
- Reaching Out: one of the characters invites the other to form a mentoring relationship. At the onset, the invitation can be met with a degree of resistance. For more on the comparison of the variants of the mentor reaching out to the mentee vs the mentee reaching out to the mentor see Payr et al. (2017).
- Mentoring Contract: once the characters agree to a mentoring relationship, the “do’s and don’ts” that guide the relationship are introduced and agreed upon.
- Training: incorporates both the acquisition of the quest-related skills and mentee’s personality development related interactions.
- Active Guidance: mentor guides mentee through a challenging situation. While the Training project entails that the challenge that mentee faces is a simulation, active guidance takes place when the situation/problem that mentee is faced with is real, hence the risks presented by the situation are also real.
- Conflict: an inter-personal conflict between mentor and mentee characters designated by the mutual dissatisfaction, and one of the character’s wish to terminate the relationship. Typically in film, Conflict projects conclude either with the two characters coming to an agreement, which leads to the formation of a stronger bond, or with one of the characters parting to return later as a new opponent/enemy to their once partner (example: in Kung Fu Panda, the evil character Tai Lung used to be Shifu’s student).
- Overcoming: captures the situation when one of the participants of mentoring relationship feels overwhelmed by the challenges presented by the quest and doubts their ability to succeed in it while the other character provides encouragement and emotional support in order to prevent the partner from giving up and quitting.
- Counseling: mentee character approaches the mentor with an urgent question/problem that the mentor helps to address. Counseling is not to be confused with the Reaching out project, as the former takes place when the mentoring relationship is already formed, and both characters have an explicit interest in resolving the problem.
- Bonding: an emotional exchange between the characters that leads to strengthening of their interpersonal relationship.
- Gifting: Gifting is similar to Bonding in terms of the role it plays for the psycho-social dynamics of mentoring relationship. Gifting is structured around one or both characters presenting the other with a gift that is symbolic of the quest (e.g. new surfing board in Chasing Mavericks), and serves to signal the partner that their value extends beyond the functional role they play in the quest.
- Exposure: Exposure is the project that concludes mentee’s training. The mentee is presented with an opportunity to show their skills in a real challenge while the mentor either participates in the challenge alongside the mentee or assumes the position of an outside observer.
- Initiation: mentor announces that mentee has completed their traineeship. The occasion is marked by the ceremony of initiation or by mentor revealing to mentee the Truth about the quest.
- Sacrifice: one of the characters offers to sacrifice themselves so that others can escape. Typically, other characters would attempt to talk them out of it, but eventually agree and leave them to face the enemy forces. More often than not, Sacrifice results in mentor’s death.
- Separation: not to confuse with the separation relationship phase that is typically marked by a conflict and temporary distancing of the two characters. During the Separation project, mentor acknowledges that there is nothing else they can teach the mentee, hence the latter must continue on their path alone. A more dramatic scenario of the Separation might also involve the mentor’s death at the hands of the villain.

Similarly, to intents, manual annotation for projects is not a straightforward task. For instance, in many cases the boundaries of the projects are not clear-cut. Even in scripted dialogues where the necessity of advancing the narrative introduces additional structural constrains (i.e. a scripted character is less likely to “bury” or abandon a project in contrast to a participant in a natural conversation), different projects might overlap, or the characters can carry out more than one project simultaneously. For example, *Overcoming*, the project that evolves around an event of mentee experiencing doubts in their ability to succeed and mentor encouraging and supporting the mentee, can also incorporate elements of *Training*. The following scene from Kung Fu Panda illustrates how the Overcoming project is concluded with the mentor’s words of wisdom, which are typically characteristic of Training projects:

**Table:** 

O	I see you have found the Sacred Peach Tree of Heavenly Wisdom.
P	Is that what this is?
P	I am so sorry.
P	I thought it was just a regular peach tree.
O	I understand.
O	You eat when you are upset.
P	Upset?
P	I’m not upset.
P	What makes you think I’m upset?
O	So why are you upset?
O	I probably sucked more today than anyone in the history of kung fu, in the history of China, in the history of sucking.
O	Probably.
P	And the Five man, you should have seen them, they totally hate me.
O	Totally.
P	How’s Shifu ever going to turn me into the Dragon Warrior?
P	I mean, I’m not like The Five.
P	I’ve got no claws, no wings, no venom.
P	Even Mantis has those thingies.
P	Maybe I should just quit and go back to making noodles.
O	Quit, don’t quit.
O	Noodles, don’t noodles.
O	You are too concerned with what was and what will be.
O	There is a saying: Yesterday is history, tomorrow is a mystery, but today is a gift.
O	That is why it is called the present.

In order to address this limitation, for the manual annotation the following guidelines were formulated: i. Project labels are to be decided upon based on the dominant theme/process manifested in the sequence at hand and ii. Seeing that film scenes frequently evolve around key events/transitions, the boundaries of the scenes can be assumed to coincide with the boundaries of projects.

### Relationship Phases

Lastly, the macro-level structural category – relationship phases – was adopted from the seminal work of Kathy E. Kram on mentoring in professional organizations (Kram 1983). With an aim to describe how interpersonal dynamics in mentoring relationship develops over time, based on qualitative research, Kram proposed to distinguish between four mentoring relationship phases: initiation, cultivation, separation, and redefinition. *Initiation phase* is characterized by mentee’s heightened respect and admiration for the mentor. At the same time, for the mentor, the mentee represents the potential that they can help to unravel. Career- and personal development-oriented processes peak at the *Cultivation phase*. As mentor and mentee get to know each other better, the expectations that they had at the onset of their relationship are tested against reality, and the true value of each individual is revealed to the other. The *Separation phase* is marked by intensified, oftentimes negative, emotional experiences, anxiety and feeling of loss, and results in the reassessment of the relationship by both participants. With increased autonomy, the mentee shows resistance towards the mentor, while the mentor may feel resentful, also in light of the recognition that the mentee is soon to move on. If separation phase does not lead to a premature termination of the mentoring relationship, the relationship normally transitions into *Redefinition phase*. At this point, the relationship is typically more egalitarian; mentor continues to support and occasionally counsel mentee, but in the capacity of a friend.

The relationship phases, as outlined by Kram, map well on to the overall structure of scripted mentoring relationships. Fictional mentorship will also undergo an initiation, when the two characters meet for the first time and agree, sometimes after initial resistance (we will return to this point in the following chapters) to form a mentoring relationship. Initiation will always be followed by an extensive cultivation period, after which an intrapersonal or interpersonal crisis will commonly ensue. Finally, the relationship will transition in to redefinition phase, at which point mentee typically faces the ultimate challenge they were training for, while their relationship with the mentor either terminates (due to mentor’s death or departure) or evolves into friendship.

The data set was annotated for relationship phases manually. At this stage, we relied upon identifying and clustering events and processes characteristic of different mentoring relationship phases as described by Kram. In Results, we will discuss the composition of relationship phases in terms of projects and intents in more details.

## Results and Interpretation

### Intents

Quantitative analysis of mentor intents (Table 3) shows the strong prevalence of Explain intent overall and per mentor character. The second most frequent intent used by mentors is Instruct, followed by Encourage, Ask, Social, Evaluate, and Single out. For mentees, in overall count, Ask, Resist, Doubt, Respond, Social, and Confirm are among the most frequent intents engaged.

**Table 3.** Value counts for Mentor and Pupil intents.

Mentor			Pupil		
Intent	*N*	%	Intent	*N*	%
Explain	188	0.28	Ask	73	0.14
Instruct	93	0.14	Resist	56	0.11
Encourage	33	0.05	Doubt	55	0.10
Ask	30	0.05	Respond	50	0.10
Social	30	0.05	Social	47	0.09
Evaluate	30	0.05	Confirm	46	0.09
Single out	30	0.05	Excitement	32	0.06
Test	29	0.04	Self-affirmation	32	0.06
Confirm	27	0.04	Report	23	0.04
Support	26	0.04	Explain	18	0.03
Dismiss	22	0.03	Share	16	0.03
Scold	20	0.03	Surprise	10	0.02
Respond	19	0.03	Apologize	9	0.02
Set the rules	15	0.02	Challenge	7	0.01
Profile	12	0.02	Problem setting	7	0.01
Seek out	12	0.02	Praise	6	0.01
Share	10	0.02	Reinforce	6	0.01
Seek confirmation	8	0.01	Test	5	0.01
Apologize	6	0.01	Support	5	0.01
Problem setting	6	0.01	Instruct	5	0.01
Resist	5	0.01	Na	4	0.01
Gift	4	0.01	Volunteer	3	0.01
Departing	3	0.00	Seek feedback	3	0.01
Volunteer	3	0.00	Seek out	2	0.00
Confusion	2	0.00	Gratitude	2	0.00
Excitement	1	0.00	Confusion	1	0.00
Seek encouragement	1	0.00	Seek Encouragement	1	0.00
–	–	–	Dismiss	1	0.00

Per mentor (Table 4), the results for Oogway reveal the significant prevalence of the intents oriented toward psycho-social dimension of mentoring (e.g. Encourage and Support), while intents typical of initiation and cultivation relationship phases (e.g. Seek out, Set the rules, Test) are not manifested. Oogway, a supporting senior mentor character in Kung Fu Panda, is introduced in the script as a participant of the already pre-exisiting mentoring relationship with the lead mentor character Shifu. Hence, we can assume that, though not explicitly addressed in the script, the two characters had already undergone initiation, cultivation and, possibly, separation phases. Hence, we encounter them at redefinition phase which is characterized by a more peer-like social dynamics and corresponding intents structure.

**Table 4.** Value counts for mentors intents.

	Frosty		Oogway		Shifu		Morpheus	
Intent	*N*	%	*N*	%	*N*	%	*N*	%
Apologize	–	–	–	–	5.0	0.03	1.0	0.00
Ask	10.0	0.05	3.0	0.05	7.0	0.05	10.0	0.04
Confirm	7.0	0.03	6.0	0.11	7.0	0.05	7.0	0.03
Confusion	–	–	–	–	2.0	0.01	–	–
Departing	–	–	2.0	0.04	1.0	0.01	–	–
Dismiss	13.0	0.06	–	–	9.0	0.06	–	–
Encourage	4.0	0.02	7.0	0.12	16.0	0.10	6.0	0.03
Evaluate	13.0	0.06	1.0	0.02	14.0	0.09	2.0	0.01
Excitement	–	–	–	–	1.0	0.01	–	–
Explain	48.0	0.22	15.0	0.27	23.0	0.15	102.0	0.43
Gift	2.0	0.01	–	–	2.0	0.01	–	–
Instruct	32.0	0.15	1.0	0.02	21.0	0.14	39.0	0.16
Problem setting	–	–	1.0	0.02	2.0	0.01	3.0	0.01
Profile	7.0	0.03	–	–	2.0	0.01	3.0	0.01
Resist	1.0	0.00	–	–	4.0	0.03	–	–
Respond	5.0	0.02	4.0	0.07	5.0	0.03	5.0	0.02
Scold	11.0	0.05	–	–	9.0	0.06	–	–
Seek confirmation	7.0	0.03	–	–	1.0	0.01	–	–
Seek encouragement	1.0	0.00	–	–	–	–	–	–
Seek out	1.0	0.00	–	–	3.0	0.02	8.0	0.03
Set the rules	7.0	0.03	–	–	1.0	0.01	7.0	0.03
Share	10.0	0.05	–	–	–	–	–	–
Single out	3.0	0.01	4.0	0.07	4.0	0.03	19.0	0.08
Social	8.0	0.04	5.0	0.09	3.0	0.02	14.0	0.06
Support	8.0	0.04	7.0	0.12	1.0	0.01	10.0	0.04
Test	19.0	0.09	–	–	9.0	0.06	1.0	0.00
Volunteer	–	–	–	–	3.0	0.02	–	–

While all mentors (with exception of Oogway for the reasons we discussed above) predominantly rely on Explanation and Instruction to carry out their mentoring function, the observed variation in the distribution of the remaining intents hints at the differences in personalities and mentoring styles of the three characters in question. For example, Morpheus, identified in our prior research as an archetypical mentor (Payr et al. 2017), is less prone to Scold, Test and Evaluate his mentee comparing to Frosty and Shifu. At the same time, this character is more likely to Single Out his mentee. Meanwhile, Frosty is the only mentor in our sample who manifests Share and Seek Encouragement intents. We hypothesize that such intents distribution is more characteristic of *relational mentoring* – a type of mentoring, wherein both participants learn and grow mutually (Hudson and Millwater 2008). In contrast, Shifu and Morpheus represent a more traditional mentoring paradigm, wherein emphasis is placed primarily on the guidance that mentors provide to their mentees, and less so on the processes of change undergone by mentors.

Per mentee (Table 5), contrary to the expected, the distribution of intents shows more variation when compared to mentors. For example, Jay relies on Confirm and Respond intents, as well as a higher number of Share intent which supports our hypothesis that Chasing Mavericks portrays relational, rather than traditional mentorship. The prevailing intent for Po from Kung Fu Panda is Doubt, followed closely by Excitement, Self-Affirmation and Social. Neo, on the other hand, relies on Asking and, similarly to Jay, Responding, with Doubt as the close third.

**Table 5.** Value counts for pupils intents.

	Jay		Shifu		Po		Tai Lung		Neo	
Intent	*N*	%	*N*	%	*N*	%	*N*	%	*N*	%
Apologize	3.0	0.02	–	–	6.0	0.02	–	–	–	–
Ask	11.0	0.09	10.0	0.18	20.0	0.08	3.0	0.14	29.0	0.36
Challenge	–	–	–	–	–	–	7.0	0.33	–	–
Confirm	16.0	0.13	3.0	0.05	18.0	0.07	2.0	0.10	7.0	0.09
Confusion	1.0	0.01	–	–	–	–	–	–	–	–
Dismiss	–	–	1.0	0.02	–	–	–	–	–	–
Doubt	3.0	0.02	3.0	0.05	38.0	0.15	–	–	11.0	0.14
Excitement	–	–	–	–	30.0	0.12	–	–	2.0	0.03
Explain	3.0	0.02	4.0	0.07	7.0	0.03	–	–	4.0	0.05
Gratitude	2.0	0.02	–	–	–	–	–	–	–	–
Instruct	2.0	0.02	3.0	0.05	–	–	–	–	–	–
Na	–	–	–	–	4.0	0.02	–	–	–	–
Praise	2.0	0.02	–	–	2.0	0.01	–	–	2.0	0.03
Problem setting	–	–	7.0	0.13	–	–	–	–	–	–
Reinforce	3.0	0.02	–	–	2.0	0.01	–	–	1.0	0.01
Report	15.0	0.12	1.0	0.02	4.0	0.02	–	–	3.0	0.04
Resist	10.0	0.08	16.0	0.29	22.0	0.09	3.0	0.14	5.0	0.06
Respond	15.0	0.12	1.0	0.02	20.0	0.08	2.0	0.10	12.0	0.15
Seek encouragement	–	–	–	–	1.0	0.00	–	–	–	–
Seek feedback	2.0	0.02	–	–	1.0	0.00	–	–	–	–
Seek out	1.0	0.01	1.0	0.02	–	–	–	–	–	–
Self-affirmation	3.0	0.02	–	–	27.0	0.11	2.0	0.10	–	–
Share	14.0	0.11	–	–	2.0	0.01	–	–	–	–
Social	10.0	0.08	5.0	0.09	28.0	0.11	2.0	0.10	2.0	0.03
Support	5.0	0.04	–	–	–	–	–	–	–	–
Surprise	–	–	–	–	8.0	0.03	–	–	2.0	0.03
Test	–	–	–	–	5.0	0.02	–	–	–	–
Volunteer	1.0	0.01	–	–	2.0	0.01	–	–	–	–

Notice that Table 5 also includes Shifu who, as mentioned previously, performs two roles – that of a mentor of Panda and that of a mentee of Master Oogway. Recall that we get acquainted with Oogway and Shifu at redefinition phase of their mentoring relationship, when Shifu turns to Oogway for counseling and advice (also reflected in Problem Setting intent count). However, Problem Setting comes third after Resist. Surprising at first glance, the prevalence of Resist intent for the given character can be justified if we take a closer look at the story line. In the script, Shifu is initially resistant to the idea that the clumsy Panda is the one who is chosen by Oogway to face evil Tai Lung, which explains the prevalence of Resist intent, otherwise atypical for Redefinition phase.

Interestingly, Shifu is not the only mentee to show resistance to their mentor. Though to a lesser degree, Jay, Po, and Neo also have Resist among top five most frequent intents. In the course of the qualitative analysis of the data, we identified two primary forces behind mentees’ resistance: i) resistance to the way that the mentor (mis-)treats and challenges them on the personal level and ii) quest-related crisis, when mentee loses faith in their abilities and/or mentor’s ability to train them, and wishes to abandon the quest.

The reader will notice that Table 5 also contains Tai Lung. Though not a student of Shifu and Oogway anymore, we nevertheless decided to include this character as he represents the mentoring relationship that terminates at the Separation phase with an unresolved conflict. This is reflected in the intents distribution, where Challenge constitutes a third of the overall intents per given mentee.

### Adjacency Pairs of Intents

Tables 6 and 7 provide an overview of the adjacency pairs initiated by mentees and mentors, correspondingly, as grounded in our data set. The inputs on the left side of each section represent the first part of an adjacency unit, and the inputs on the right are the candidate responses (second part of adjacency pair), followed by their frequency distribution. For example, Apologize, as the first pair part initiated by mentee, is matched by mentor’s Support as the second pair part. Meanwhile, for the first pair part Doubt, there eight response candidates are possible: Encourage, Instruct, Respond, Single Out, Support, Confirm, Explain, and Ask, in order of their frequency distribution. Note that the resulting adjacency pairs do not represent the ground truth that is valid for all instances of scripted mentor-mentee communication. A different data set may and, most likely will, reveal additional response candidates and/or variant frequency distribution.

**Table 6.** Adjacency pairs: pupil to mentor.

Pupil intent	Mentor intent	Freq.	Pupil intent	Mentor intent	Freq.
Apologize	Support	1.00	Excitement	Instruct	1.00
Ask	Explain	0.35	Excitement → Ask	Scold	1.00
	Respond	0.21	Gratitude	Encourage	1.00
	Confirm	0.10	Praise	Seek out	1.00
	Instruct	0.08	Problem setting	Explain	0.50
	Volunteer	0.04		Confirm	0.50
	Single out	0.04	Report	Explain	0.67
	Dismiss	0.04		Evaluate	0.33
	Test	0.04	Resist	Explain	0.38
	Social	0.02		Confirm	0.12
	Set the rules	0.02		Ask	0.12
	Problem setting	0.02		Respond	0.12
	Ask	0.02		Test	0.12
Challenge	Encourage	0.33		Social	0.12
	Resist	0.33	Respond	Set the rules	0.67
	Dismiss	0.33		Explain	0.33
Dismiss	Explain	1.00	Seek encouragement	Encourage	1.00
Doubt	Encourage	0.20	Seek feedback	Evaluate	0.67
	Instruct	0.20		Dismiss	0.33
	Respond	0.20	Seek out	Dismiss	0.67
	Single out	0.13		Encourage	0.33
	Support	0.07	Self-affirmation	Explain	0.67
	Confirm	0.07		Dismiss	0.33
	Explain	0.07	Social	Social	0.50
	Ask	0.07		Dismiss	0.25
Support	Seek encouragement	0.50		Scold	0.25
	Resist	0.50	Volunteer	Ask	1.00
Surprise	Ask	1.00	Surprise → Ask	explain	1.00

**Table 7.** Adjacency pairs: mentor to pupil.

Mentor intent	Pupil intent	Freq.	Mentor intent	Pupil intent	Freq.
Apologize	Resist	1.00	Instruct → encourage	Doubt	1.00
Ask	Respond	0.71	Instruct → evaluate → ask	Report	1.00
	Explain	0.12	Instruct → explain	excitement	0.50
	Confirm	0.12		Doubt	0.50
	Doubt	0.06	Instruct → explain → seek conf.	Confirm	1.00
Departing	Resist	1.00	Profile	Respond	0.78
Dismiss	Respond	0.50		praise	0.22
	Ask	0.50	Respond	Challenge	1.00
Dismiss → seek confirmation	Confirm	1.00	Scold	Apologize	0.29
Encourage	Resist	0.29		Respond	0.29
	Confirm	0.29		Resist	0.14
	Ask	0.14		Surprise	0.14
	Excitement	0.14		Social	0.14
	Doubt	0.14	Seek out	Doubt	0.50
Encourage → instruct	Surprise	1.00		Confirm	0.50
Evaluate	Resist	0.27	Set the rules	Apologize	0.50
	Respond	0.18		Doubt	0.50
	Self-affirmation	0.18	Set the rules →ask	Confirm	1.00
	Ask	0.09	Share	Ask	0.50
	Report	0.09		Social	0.50
	Doubt	0.09	Single out	Ask	0.33
	Confirm	0.09		Doubt	0.33
Eval. → apologize → enc.	Respond	1.00		Resist	0.17
Explain	Ask	0.29		Social	0.17
	Doubt	0.21	Single out → problem setting	Ask	1.00
	Resist	0.14	Single out → profile	Respond	1.00
	Surprise	0.07	Social	Ask	0.33
	Report	0.07		Social	0.33
	Respond	0.07		Respond	0.17
	Reinforce	0.07		Confirm	0.17
	Confirm	0.07	Support	Respond	0.25
Explain → instruct	Confirm	1.00		Resist	0.25
Explain → scold	Confirm	1.00		Ask	0.25
Explain → seek confirm	Confirm	1.00		Confirm	0.25
Explain → share	Confusion	1.00	Test	Resist	0.45
Explain → single out	Respond	1.00		Respond	0.27
Gift	Ask	0.50		Ask	0.09
	Social	0.50		Report	0.09
Instruct	Ask	0.40		excitement	0.09
	Social	0.20	Test → explain	Confirm	1.00
	Confirm	0.20	Test → instruct → eval. → expl.	Respond	1.00
	Report	0.10	Volunteer	Ask	0.50
	Doubt	0.10		Confirm	0.50

### Projects

**Figure 1.**
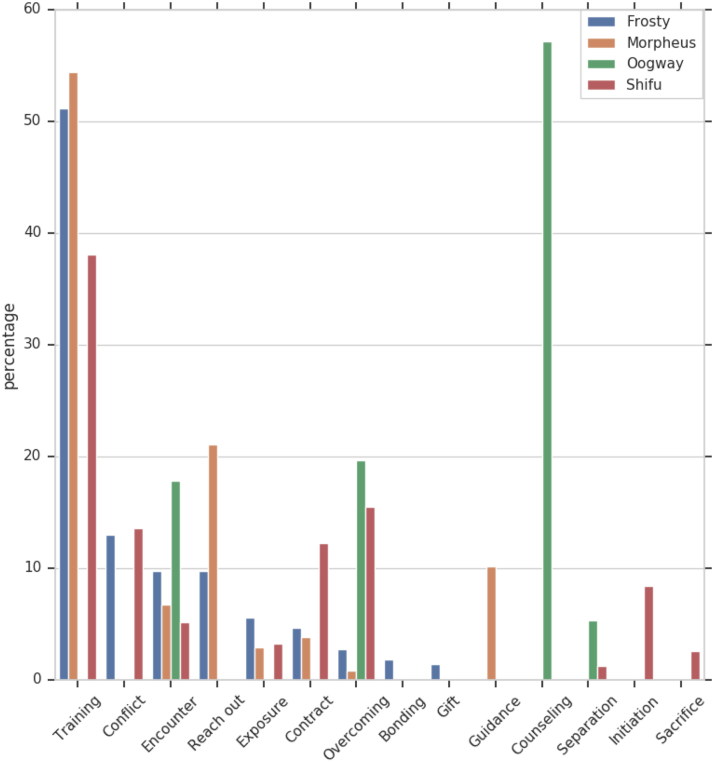
Mentors project.

**Figure 2.**
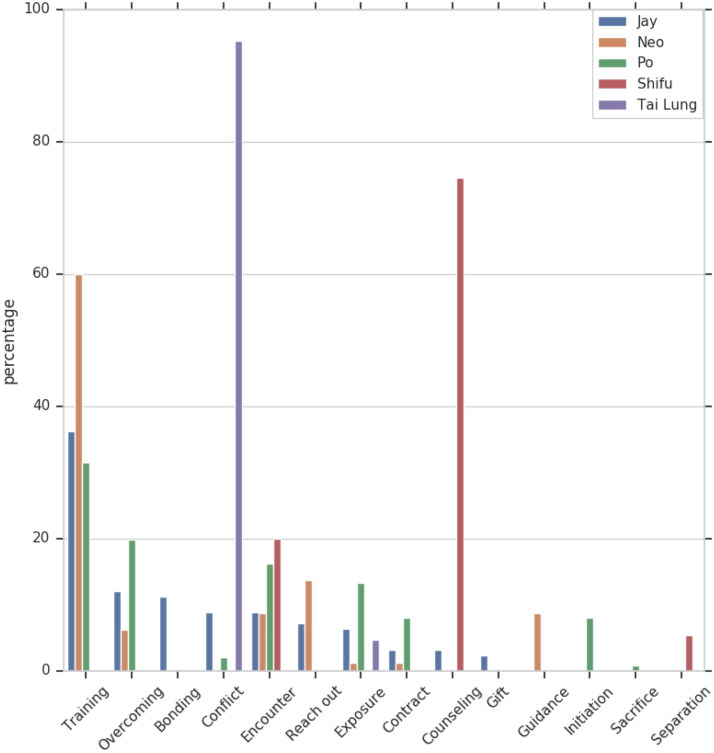
Pupil projects.

Figures 1 and 2 provide an overview of the relative length of the projects, that is the number of utterances constituting a project in relation to the overall number of utterances uttered by a character. As expected, most of the utterances of Frosty, Morpheus, and Shifu are uttered within Training projects. Recall that Oogway plays a secondary role as a mentor to Shifu, the lead mentor character. Thus, it is not surprising that most of the communication activity performed by Oogway is associated with the Counseling project.

It is important to recognize that the length and frequency of a project do not necessarily correlate with the significance of the given project within a mentoring relationship. For example, Gift in Chasing Mavericks is one of the shortest projects that takes place only once. Nevertheless, gift-giving is an important symbolic event that signifies the trust and the value that the participants of a mentoring relationship place in each other. This agrees with accounts of mentoring in real life, where gift-giving is spoken of as an act of affection that serves to deepen participants’ commitment to the other person (Lucas 2001).

**Figure 3.**
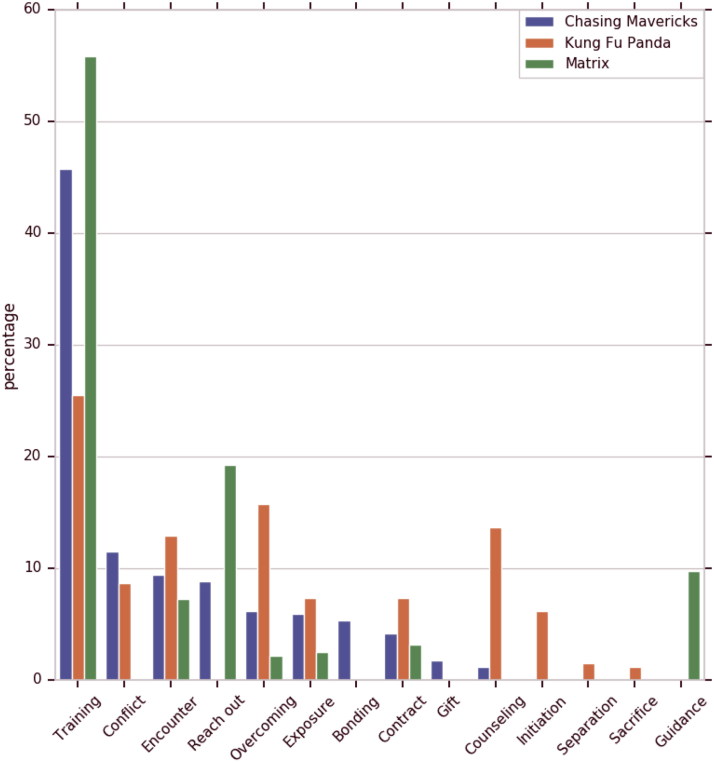
Mentor and pupil projects.

Setting aside Oogway, the projects that all mentors in our sample participate in are, in chronological order, Encounter, Mentoring Contract, Training, Overcoming, and Exposure. This is, predictably, mirrored in the projects for their respective mentees – Jay, Neo, and Po (Figures 2 and 3). Further on, the mentor–mentee pairs from Chasing Mavericks and Kung Fu Panda also participate in Conflict, while the mentor–mentee pair from Matrix does not. We cannot say conclusively whether conflict manifestation is associated with the type of relationship represented (relational mentoring v traditional mentoring), or with the film genre (drama v action film), or a third factor. That said, we believe that the role and the situational context of conflicts in mentoring relationships is an interesting and important topic that warrants further research.

### Relationship Phases

**Figure 4.**
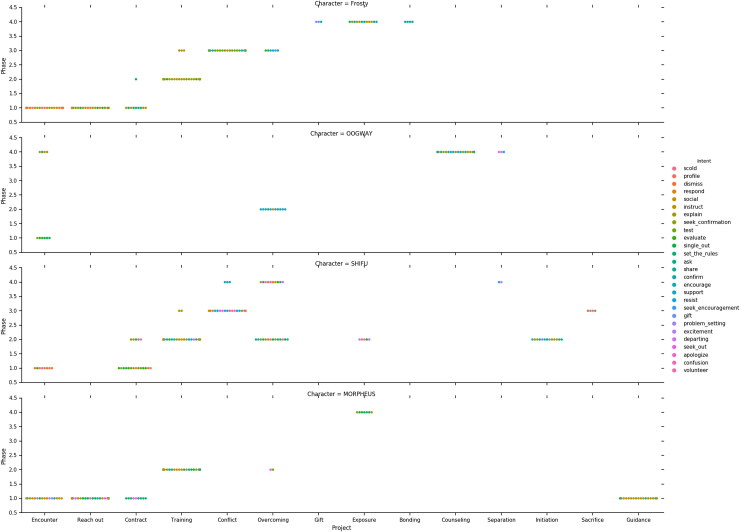
Mentors Intent and Projects in different phases. Phases mapping – 1: Initiation, 2: Cultivation, 3: Separation, 4: Redefinition.

**Figure 5.**
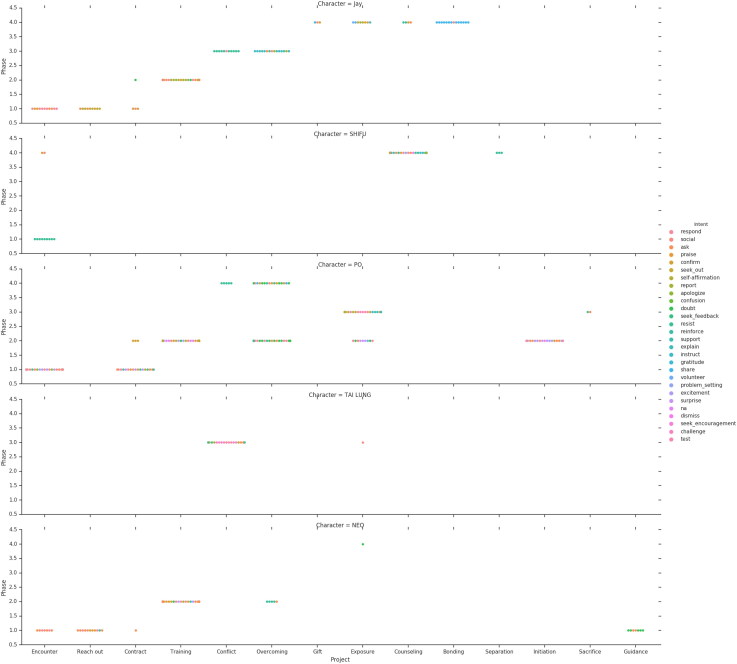
Pupils Intent and Projects in different phases. Phases mapping – 1: Initiation, 2: Cultivation, 3: Separation, 4: Redefinition.

Per Figures 4 and 5, Initiation phase is constituted by the following projects: Encounter, Reaching out, and Mentoring contract. In instances when it is the mentee who seeks out the mentor (e.g. Chasing Mavericks), Initiation phase will be characterized, among other, by Dismiss intents for mentors and Seek Out and Respond intents for mentees. Such intents composition can be explained by the behavioral pattern commonly ascribed to fictional characters. Namely, mentee is represented as the one doing the job of convincing their potential mentor to take them on as students, while mentor customarily shows initial resistance, but, having acquired more information regarding their prospective student, eventually agrees. To the contrary, in the situations when the mentor is the one reaching out to the mentee (e.g. Matrix), intents composition will include more Single out and Explain intents, owning to the mentor’s attempt to convince their mentee-to-be to join the quest. In such cases, the intents structure for mentee will be dominated by Ask and Doubt.

**Figure 6.**
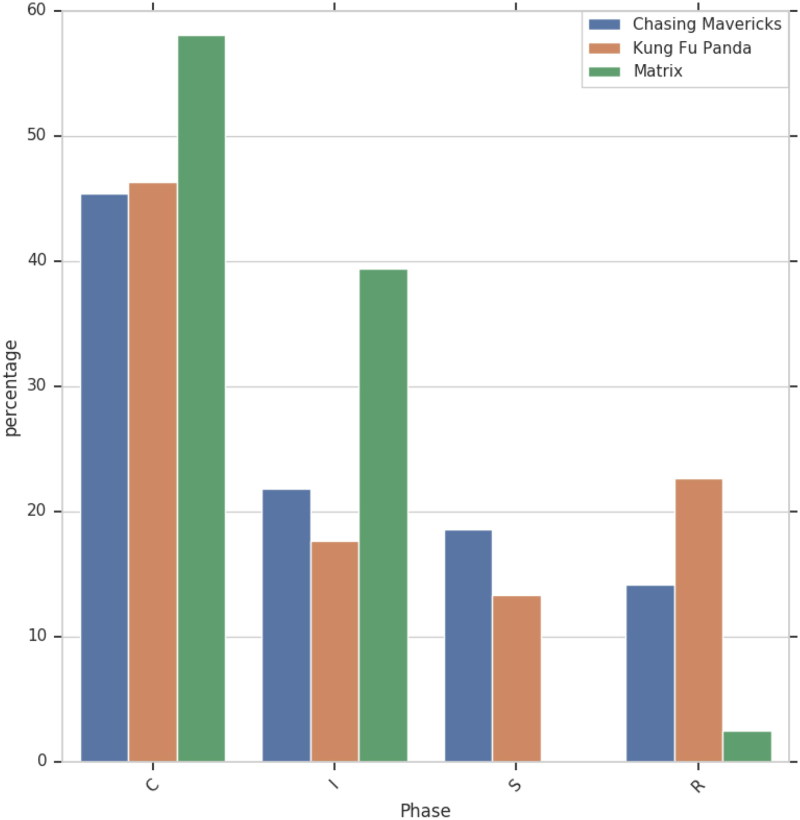
Mentor and pupil phases in scripts.

Cultivation, predictably the most lengthy phase in terms of the number of utterances that compose it (Figure 6), is commonly offset by Mentoring Contract project. An exception is Matrix, where Mentoring Contract takes place toward the end of Initiation phase. Recall that in Matrix, mentor character is the one who seeks out the mentee. In this case, the underlying objective of Mentoring contract is not to set the rules for the mentee to abide to, but to inform the mentee about the implications that will follow should they choose to participate in the quest. It is only upon mentee’s agreement that mentor can proceed to train the mentee (Cultivation phase). To the contrary, in scripts, where mentee is the one to reach out to mentor (Chasing Mavericks), or when mentee is assigned to mentor by a third party (Kung Fu Panda), Mentoring Contract establishes the do’s and don’ts of the training process and is therefore associated with the Cultivation phase.

Similarly to Separation phase in real-life mentoring relationships (Kram 1983), Separation in film is typically demarcated by one or more Conflict and Overcoming projects. In Kung Fu Panda, Conflict at Separation phase plays out between mentor Shifu and his former student Tai Lung. In Chasing Mavericks, two distinct Conflict projects take place: the interpersonal conflict that ensues Frosty’s negative feedback provided to Jay on his essay task, and the emotional turmoil that follows Frosty challenging Jay to face the situation with his missing father. In terms of the intents composition, separation phase for mentors is dominated by Test, Support, Scold, Apologize and Resist intents. For mentees, the intents composition most frequently features Resist, Challenge, Self-Affirmation, Explain and Respond intents.

Finally, Redefinition phase, as grounded in our data set, incorporates Gift, Exposure, Counseling and Bonding projects for Chasing Mavericks, Counseling, Separation, Conflict, Overcoming for Kung Fu Panda, and Exposure for Matrix. Note the absence of Exposure project in case of Kung Fu Panda, despite the fact that in the script the Panda will face and beat Tai Lung in the final battle. However, Po and Tai Lung’s encounter takes place once both Oogway and Shifu have departed. Thus Exposure in Kung Fu Panda, though a part of the fabula (Bordwell 2013), formally takes place outside of the mentoring relationship, and therefore is not included in our final data set. The events of the Oogway and Shifu’s departures constitute the Separation project (not to be confused with the Separation phase) in Kung Fu Panda. Understandably, in real life mentoring, the occurrences of mentors departing or dying at the hands of villains are rare. In film, on the other hand, said dramatic twists are common as they are dictated by the necessity to advance the narrative and enhance viewers’ engagement.

## Intent classification

In the intents classification task, our objective was to investigate the upper-limit which the automated classification methods can achieve when considering information from the utterance level only. For this purpose, we explored a range of state-of-the-art universal word and sentence embeddings pre-trained on large corpora: FastText (Joulin et al. 2016), InferSent (Conneau et al. 2017), Skip-Thought Vectors (Kiros et al. 2015), Sent2Vec (Pagliardini et al. 2018) and compared them to the baseline BOW and TFIDF based representations. Based on the obtained representations, we trained and evaluated a set of classifiers typically applied in text classification tasks. These include Support Vector Machines (Cortes and Vapnik 1995) with linear and rbf kernels, and Online Passive-Aggressive algorithm (Crammer et al. 2006), Gradient Boosting (Friedman 2001), Ada Boost (Hastie et al. 2009), Random Forests (Breiman 2001) classifiers implemented in the scikit-learn library (Pedregosa et al. 2011) with the default parameters.

For the training of the automatic classification system, 8 types of intents, which have at least 50 instances in the introduced data set, were considered: Ask, Confirm, Doubt, Explain, Instruct, Resits, Respond and Social. The total number of instances in the resulting training set is 742. Table 8. presents the best performing classifier for each distinct representation used, computed using a stratified split in ten-fold cross-validation on the training set. Performance is expressed as the average weighted F1 scores. As the results demonstrate, the classifier trained and evaluated on the Skip-Thought (ST) model based representation achieved the highest score of 0.55 offering an improvement over other state-of-the-art models.

**Table 8.** Intent classification: representations, models F1 scores.

Representation	Dim.	Dataset, model	F1 score
BOW	1420	–	0.42
TFIDF	1420	–	0.42
Sent2Vec	700	Book Corpus, unigram (Zhu et al. 2015)	0.47
InferSent	4096	Common Crawl, Glove 840B Pennington et al. (2014)	0.51
FastText	300	Wikipedia (Bojanowski et al., 2016)	0.50
Skip-Thought	4800	Book Corpus (Zhu et al. 2015), uni-ski, bi-skip	**0.55**

### Evaluation of the Automatic Classification Results

To evaluate the performance of the automatic classification against a human expert in the similar setting i.e. when classification is performed without access to metafeatures (script name, character type, relationship phase, project) and contextual information (utterances that precede and follow a target utterance in a conversation), we created a test set (*N* = 205) by randomly selecting mentor and mentee utterances from the character type annotated corpora of movie scripts described in Skowron et al. (2016) and removing all the utterances that could also be found in the training set.

**Figure 7.**
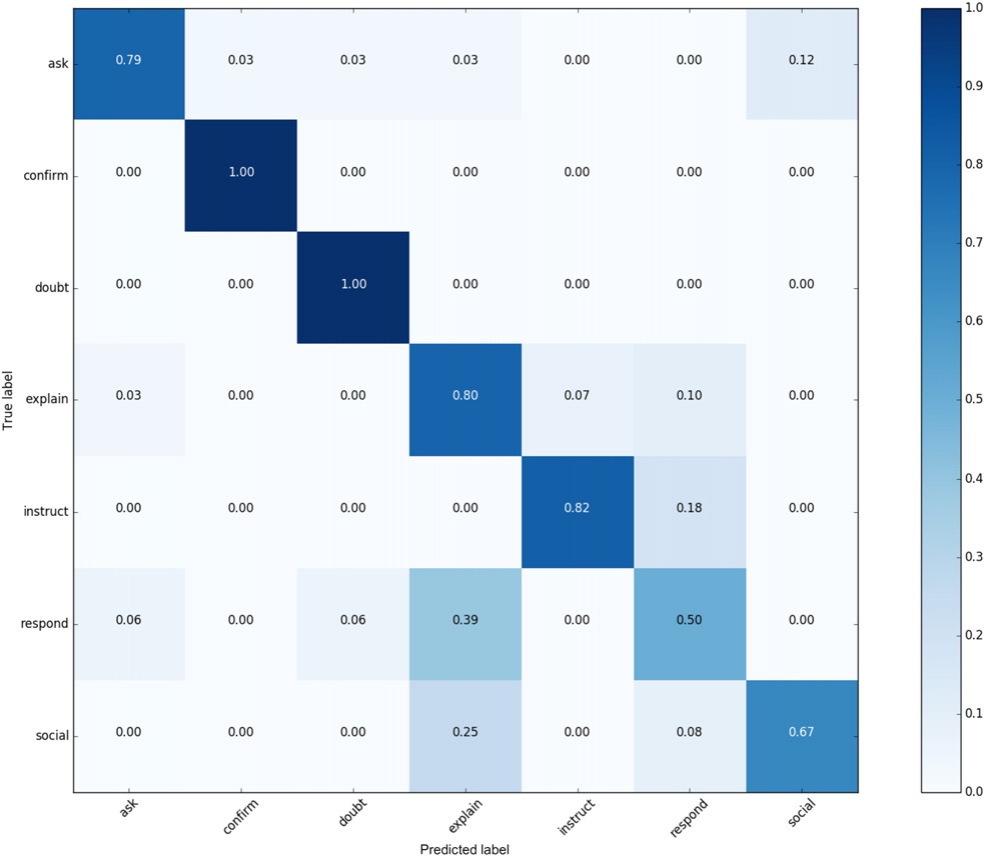
Confusion matrix for manual intent annotations: contextual information available (True label) and no-contextual information (Predicted label).

Figure 7 presents the confusion matrix between the human expert annotations in the condition when contextual information is available (True labels) against the utterance-level annotation condition (Predicted label) in the intent classification task with 8 distinct labels. Based on the data obtained, we can infer that interpretation of Social, Respond and Ask intents is the most sensitive to contextual factors. Frequent confusion between Respond and Explain intents can be justified by the narrative form (“tell about”) (Payr et al. 2017) that both intents typically take. In the context-free annotation scenario, it is hard to infer whether a dominant function that a narrative unit performs is to explain something pertaining the training domain, or whether given narrative unit is a response to a mentee’s question. Matters complicate if we consider that activities of responding and explaining can manifest simultaneously.

To enable the comparison between the automatic classification results conducted on features representing utterance content, we calculated the F1 score for the labels annotated without the contextual information, using the annotations by a human expert with the contextual information available as the ground truth. The resulted F1 score of 0.75 can be used to express an estimation for the upper limit of (human) performance when contextual information is not provided, and also sets the bonds to the performance of the automatic classification without context information.

**Figure 8.**
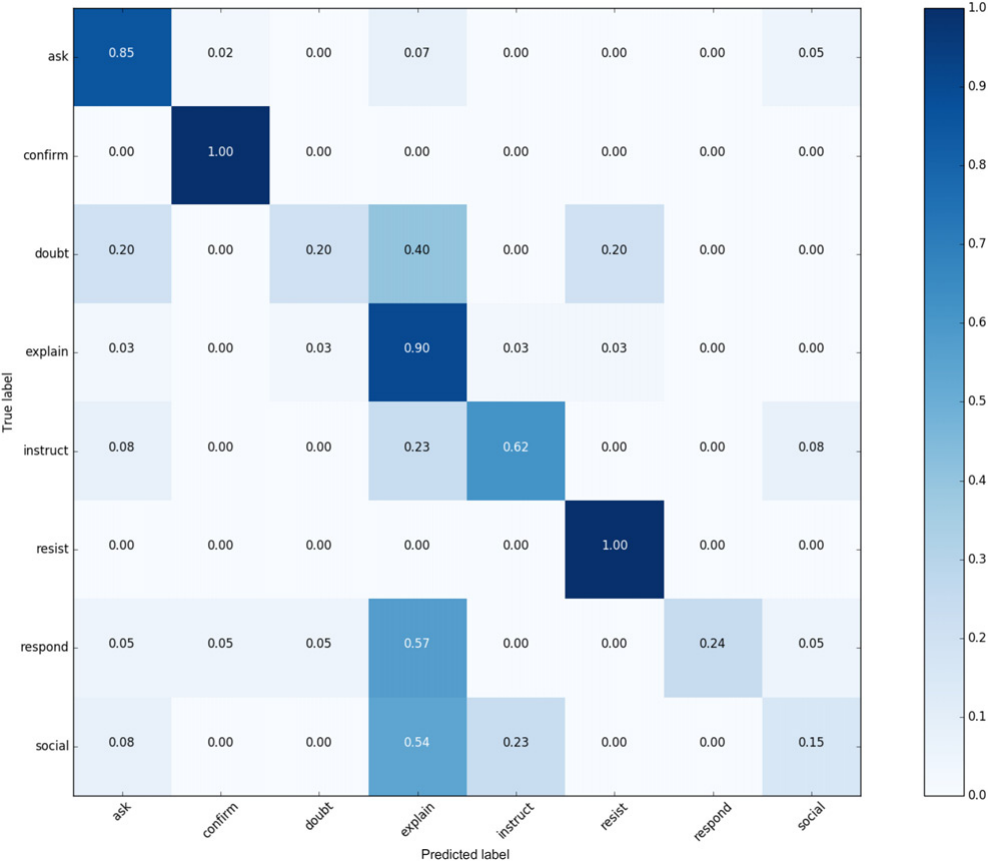
Confusion matrix for automatic intent classification.

Furthermore, the developed test set was used to evaluate the performance of the automated intent classifier. Figure 8 presents the confusion matrix for the SVM based classifier of the best performing semantic representation (ST) on the test set.

As expected, the automated classifier performance (F1 = 0.64) in the classification task is weaker comparing to a human expert (F1 = 0.75). Interestingly, this result does not hold across all the labels. Specifically, automated classifier shows improved performance when classifying for Explain (0.9 against 0.8) and Ask (0.85 against 0.79) labels. We hypothesize that the reasons for the stronger performance of the automated classifier on these categories are the following: i.) human expert annotations (no context) was more complex (had to consider a complete set of labels, while automatic classifier was trained on a subset, 8 most frequent labels and evaluated on this set), ii.) the classifier was trained on the data set that included labels assigned to the utterances with context considered, while the initial annotation by an expert did not have such information available, iii.) differences between the data sets (realizations of labels, frequencies), and iv.) human inconsistencies.

## Discussion and Future work

The present model of the mentor-mentee conversation activities, joint goals and relationship phases can serve as a blueprint for analogous studies of the layered structure of dialogues between fictional characters at large. Such studies not only shed light on the structural components and dynamics of the goal-oriented communication, but are also helpful to support and inform the design of the automated dialogue systems. Specifically, they allow to formulate a set of constraints to direct the systems’ responses to adhere with the expectations and requirements of the conversation activity at hand. The automatic intent classifier results provide further support for this assumption and illustrate how manual text analysis approaches can be integrated with computational methods in a complimentary fashion leading to better grounded and informed modelling. It is important however to be aware of the limitations of the presented approach. Primarily, the limited data set used in the current study does not allow to make generalized conclusions regarding the content of the three categories proposed within the model. An analysis of a significantly larger corpus is desirable if one wishes to investigate robustness of the labels comprising the categories, and the factors (e.g. character type, film genre etc, type of mentoring relationship) that play a role in shaping their composition and syntax. Due to the complex and dynamical nature of human conversations, wherein the function of an utterance depends not only on its semantic theme, but its position in a conversation and a number of metafeatures, assigning category labels to utterances and their sequences is not a straightforward task. To the contrary, this task presents challenges both to human experts and automated systems. For further improvement of the automatic classification task performance, additional aspects such as contextual information, with an emphasis on the character type and the utterances surrounding the target utterance, and the upper bound of the performance of the state-of-the-art methods for the semantic utterance representation should be included. Mentoring relationships in real life vary in their quality and degree of satisfaction that participants hold (Humberd and Rouse 2016). Even though none of the mentors in our sample can be described as a straightforward manipulative mentor, or a mentor who sabotages their relationship with mentee, it is hard to judge the quality of the mentoring relationships explored; especially if we consider that in the context of a fictional story-world, the criteria for an assessment of success of a mentoring relationship (e.g. participants’ satisfaction, mentee’s career growth etc.) hardly apply. For a more realistic model of mentor-mentee dialogues, some criterion of quality of mentoring relationship would have to be considered.

## Conclusions

For the task of designing a synthetic conversational agent, it is important to recognize the key processes and sub-goals associated with a specific activity type, and how these processes manifest in language. In this paper, we presented the results of our in-depth investigation of mentoring as one example of a conversation activity type, which is especially relevant in the context of developing sector of online tutoring. Three structural categories – intents, projects, and relationship phases – operating at different levels of conversations between fictional mentors and mentees were introduced, followed by a quantitative exploration of the relationship between these categories. In addition, automated methods of classification of utterances comprising mentoring activity type were explored, and their performance compared to that of a human expert. It is our assumption that the resulting model of mentor–mentee communication can be helpful in putting forward a system of constrains that would enable a more realistic communication between a human conversant and a machine.

## Notes

1 https://www.imsdb.com/.

2 https://www.springfieldspringfield.co.uk/.
